# Interaction of Bacteria, Immune Cells, and Surface Topography in Periprosthetic Joint Infections

**DOI:** 10.3390/ijms24109028

**Published:** 2023-05-19

**Authors:** Cristina Belgiovine, Luca Pellegrino, Alberto Bulgarelli, Francesca Cecilia Lauta, Alessia Di Claudio, Roberta Ciceri, Assunta Cancellara, Francesca Calcaterra, Domenico Mavilio, Guido Grappiolo, Katia Chiappetta, Mattia Loppini, Roberto Rusconi

**Affiliations:** 1 IRCCS Humanitas Research Hospital, 20089 Rozzano, Italy; roberta.ciceri@humanitasresearch.it (R.C.); assunta.cancellara@humanitasresearch.it (A.C.); francesca.calcaterra@humanitasresearch.it (F.C.); domenico.mavilio@humanitas.it (D.M.); mattia.loppini@hunimed.eu (M.L.); roberto.rusconi@hunimed.eu (R.R.); 2 Department of Clinical, Surgical, Diagnostics and Pediatric Sciences, University of Pavia, 27100 Pavia, Italy; 3 Department of Biomedical Sciences, Humanitas University, 20072 Pieve Emanuele, Italy; luca.pellegrino@humanitasresearch.it (L.P.); alberto@bulgarelli.eu (A.B.); francesca.lauta@humanitasresearch.it (F.C.L.); alessia.diclaudio@humanitasresearch.it (A.D.C.); guido.grappiolo@humanitas.it (G.G.); katia.chiappetta@humanitas.it (K.C.); 4 Unit of Clinical and Experimental Immunology, Department of Medical Biotechnologies and Translational Medicine, University of Milan, 20089 Rozzano, Italy; 5 Fondazione Livio Sciutto Onlus, Università Degli Studi Di Genova, 17100 Savona, Italy

**Keywords:** periprosthetic joint infection (PJI), immune response, surface topography, staphylococcal biofilms

## Abstract

The incidence of periprosthetic joint infections (PJIs) is ~2% of total procedures and it is expected to rise due to an ageing population. Despite the large burden PJI has on both the individual and society, the immune response to the most commonly isolated pathogens, i.e., *Staphylococcus aureus* and *Staphylococcus epidermidis*, remains incompletely understood. In this work, we integrate the analysis of synovial fluids from patients undergoing hip and knee replacement surgery with in-vitro experimental data obtained using a newly developed platform, mimicking the environment of periprosthetic implants. We found that the presence of an implant, even in patients undergoing aseptic revisions, is sufficient to induce an immune response, which is significantly different between septic and aseptic revisions. This difference is confirmed by the presence of pro- and anti-inflammatory cytokines in synovial fluids. Moreover, we discovered that the immune response is also dependent on the type of bacteria and the topography of the implant surface. While *S. epidermidis* seems to be able to hide better from the attack of the immune system when cultured on rough surfaces (indicative of uncemented prostheses), *S. aureus* reacts differently depending on the contact surface it is exposed to. The experiments we performed in-vitro also showed a higher biofilm formation on rough surfaces compared to flat ones for both species, suggesting that the topography of the implant could influence both biofilm formation and the consequent immune response.

## 1. Introduction

Total joint replacement is a safe and common procedure that can restore function, while improving the quality of life in patients with hip and knee diseases. A prosthetic joint infection (PJI) represents one of the most frequent and severe complications after joint replacement, leading to implant failure and often surgical revision [1,2]. The most frequently isolated pathogens in PJIs are *Staphylococcus aureus* (31–43%) and coagulase-negative (CN) staphylococci, such as *Staphylococcus epidermidis* (20–31%). Depending on the time since the primary onset of the symptoms, PJI can be classified as acute (early onset: <4 weeks after implantation) or chronic (delayed onset: >4 weeks up to 3 years) [3]. Acute infections manifest themselves with rapidly increasing pain, red/swollen joints, and systemic signs of inflammation, such as fever, up to septic shock. They are caused predominantly by highly virulent pathogens (e.g., *S. aureus*, streptococci, and enterococci). Chronic infections present with subtler symptoms, such as chronic joint pain, early loosening of the prosthesis, and sinus tract. They are managed with explantation and the one- or two-stage exchange of the implant combined with appropriate antimicrobial therapy. Chronic PJIs are mostly biofilm-related infections and are usually caused by bacteria, such as CN staphylococci [4,5,6]. The complex pathophysiology of PJI is associated with the microbiological properties of bacteria and/or fungi associated with the implant [7,8,9,10]. One aspect that could impair the revision of implants after both acute and chronic infection is recurrence due to biofilm formation. Biofilms are complex communities of microorganisms that adhere to surfaces and are embedded in a self-secreted matrix of extracellular polymeric substances (EPS). Biofilm formation is a multi-step process and involves contributions from physical, biological, and chemical factors [11,12,13]. While bacteria near the surface of biofilms are active, bacteria that are embedded deeper are quiescent or in various stages of dormancy through altered gene transcription [14] or quorum sensing (QS)-signaling molecules [15]. Recent studies have shown that some bacterial QS molecules, such as acyl-homoserine lactones (AHLs), can modulate host immune responses by regulating the expression of cytokines and chemokines, which can promote or inhibit inflammation [16]. In addition, it has been shown that biofilms can skew macrophage polarization towards an anti-inflammatory phenotype, favoring bacterial persistence [17,18]. For these reasons, bacterial biofilms are known to play an important role in chronic device-related infections [19] and have been, together with their constituents, the center of interest of several trials focusing on novel approaches to fight bacterial diseases and sepsis [20].

In the evaluation of PJI onset and biofilm formation, very little is known about the role of surface topography. It is well-established that the presence of a foreign body triggers a host response [21,22] divided into four phases: (i) implantation, (ii) blood–biomaterial interaction, (iii) inflammation, (iv) and tissue remodeling [23]. This can lead to prosthetic failure and ultimately revision procedures. However, as shown for breast implants, the immune system may also be affected by different surface topographies [24]. Similarly, surface roughness and waviness have been shown to significantly affect bacterial attachment and biofilm formation [25,26].

Differences in orthopedic implant surface topography can be linked to cemented or uncemented joint replacements. Cement placement at the time of primary or revision arthroplasty provides stability, fills any gaps and irregularities, and ameliorates implant positioning. Moreover, cement acts as a physical barrier, preventing debris or particles from becoming lodged in between the prosthesis and bone and causing the loosening or failure of the implant [27,28]. However, cementation is not always necessary or appropriate in every case, and the decision to use it should be made on an individual basis by the surgeon based on various factors, such as patient age, activity level, bone quality, and the type of surgery being performed. Cementless techniques, for example, not only are associated with shorter operating times, but may be best in young subjects for reasons that are related to a better integration with bone and a higher preservation of autologous bone [29]. Indeed, the trabecular metal (TM) acetabular cup, used as reference surface is this study, is employed in cementless hip revision surgery, given its extremely high porosity (up to 85%), being able to provide attachment strengths that are 3- to 6-fold higher than in prior fibrous tissue studies using sintered beaded porous coatings [30]. Still, the risk of re-revision of TM components for infection is comparable to non-TM acetabular components [31]. Overall, the pathophysiological aspects of PJI associated with cemented or uncemented implants, and in general the role played by surface topography in promoting biofilm formation and skewing the immune response, are not known.

In this work, we show that a comprehensive understanding of periprosthetic joint infections requires an examination of the relationship between implant surface topography, bacterial colonization, and immune cell response. Our research examines the immune cell composition and cytokine release in samples from joint arthroplasties to highlight differences between pre-implant conditions and revision surgeries, whether septic or aseptic. We demonstrate the immunomodulatory effect of bacterial-specific contamination in the case of septic revisions, both systemically and in the synovial fluid. Furthermore, we introduce a novel in vitro platform that allows the investigation of biofilm formation and immune response in correlation with a specific surface topography, i.e., smooth or rough, representing the conditions of cementless or cemented implants, respectively. Our findings indicate that cementless implants tend to promote bacterial colonization and a pro-inflammatory environment in association with potential acute infections.

## 2. Results

### 2.1. Immune Response to Joint Replacement

We collected samples from 74 patients undergoing either primary joint replacement (PR) or revision surgeries at Humanitas Clinical Hospital in Rozzano (Italy) from 2016 to 2021. The clinical data for our patients are presented in Table 1.

**Figure 1:**
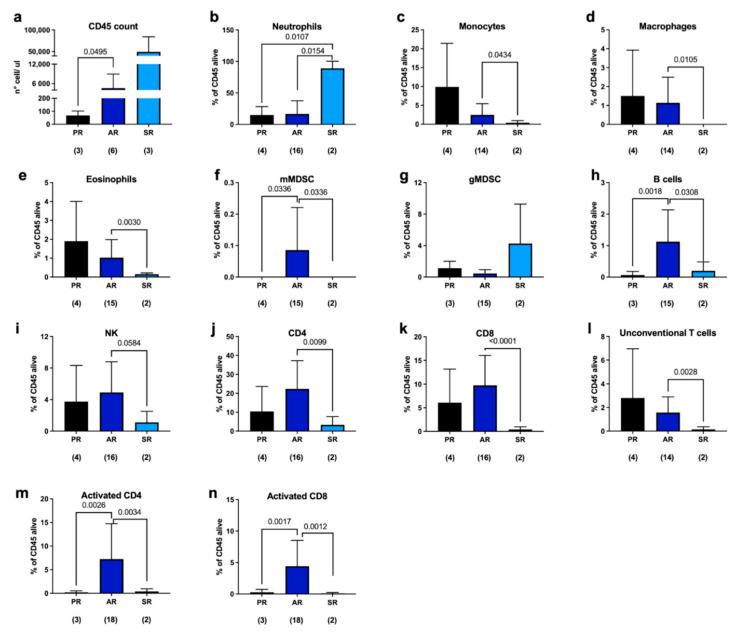
FACS analysis of periprosthetic leukocytes derived from patients undergoing either primary replacement (PR), aseptic revision (AR), or septic revision (SR). (**a**) CD45 cell count was performed using beads derived from Leukocounts kit (Becton and Dickinson, Franklin Lakes, NJ, USA) on a subset of samples. (**b**–**l**) Live cells were selected and the percentage was calculated on CD45-positive viable cells. (**m**,**n**) Analysis of T-cell activation was performed on different patients using CD69 as marker. The number below each group represents the number of samples analyzed. Histograms represent the mean ± standard deviation. Statistical analysis: unpaired *t*-test with Welch’s correction.

Fluorescence-activated cell sorting (FACS) analysis was performed to evaluate the presence and abundance of immune cells in the periprosthetic microenvironment. We observed a significant increase in the number of CD45 cells in septic patients, as expected, but, surprisingly, we also found an increase in their number in aseptic samples compared to what was found in the case of primary replacement (Figure 1a). Moreover, in septic patients, we found a considerable amount of neutrophils (Figure 1b, light blue column) that remarkably reduced the percentage of other immune populations (Figure 1c–l, light blue columns).

Regarding the periprosthetic microenvironment of patients undergoing aseptic revision (AR), we observed an increase in the following in the several populations examined in our analysis with respect to PR, although not statistically significant: myeloid-derived suppressor cells (MDSCs), natural killer (NK) cells, CD4 T-helper cells, and CD8 cytotoxic T lymphocytes (Figure 1f–k, blue bars). Monocytes, macrophages, eosinophils, and unconventional T cells were found to be reduced in aseptic patients compared to primary replacement synovial fluid; however, these differences do not appear to be significant (Figure 1c–e,l; blue bars). Notably, we observed a significant increase in the percentage of B cells, monocytic MDSCs, and CD69-positive CD4 and CD8 cells, indicating an activation of the cellular response (Figure 1i–n, blue columns). The elicitation of an adaptive immune response could be due to the initial activation of innate immune cells, resulting in the recruitment of T cells to the site of inflammation or in the release of cytokines and chemokines by innate immune cells [32,33].

**Figure 2:**
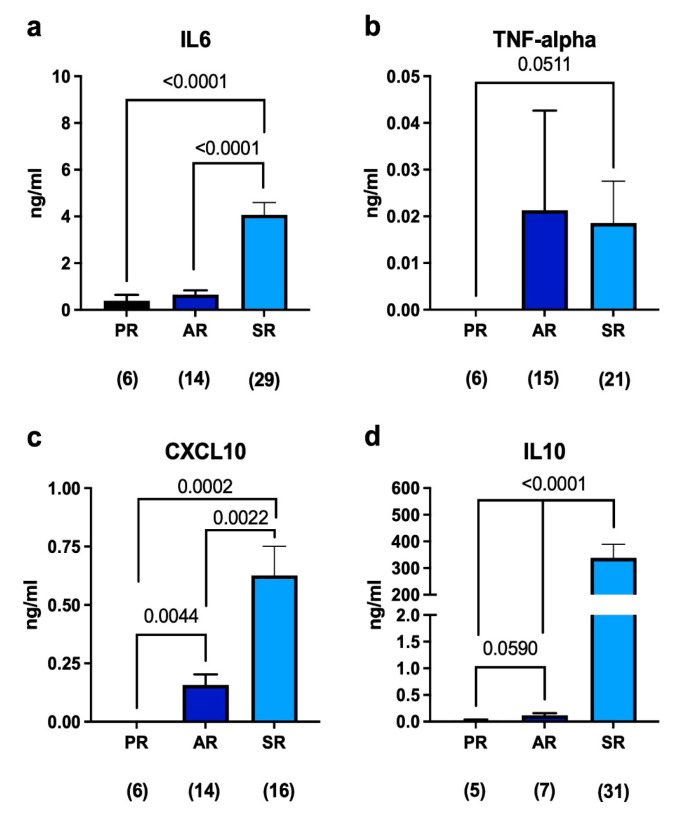
(**a**–**d**) ELISA quantification of soluble mediators in the synovial fluid of patients undergoing either primary replacement or revision surgeries. The number below each group represents the number of samples analyzed. Histograms represent the mean ± standard deviation. Statistical analysis: unpaired *t*-test with Welch’s correction.

In the synovial samples, we also quantified, via enzyme-linked immunosorbent assay (ELISA), the concentration of cytokines, such as IL-6, which is an inflammatory cytokine used for the diagnosis of PJI; TNF-alpha, which is produced more by macrophages involved in the acute phase of infection and associated with T-cell activation; CXCL10, a cytokine produced in response to interferon-gamma activation; and IL-10, which is an anti-inflammatory mediator. This analysis has shown that IL-6 was detected at very low levels in patients undergoing primary replacement and it also increased, although not significantly, in aseptic patients. In contrast, IL-6 was released in large amounts in septic patients (Figure 2a), confirming its use as a marker of infection. On the other hand, TNF-alpha was absent in the synovial fluids of patients undergoing primary replacement, but it was detected in the periprosthetic spacing of both septic and aseptic revision patients (Figure 2b). CXCL10 showed a similar behavior to IL-6, with a greater difference between aseptic revisions and primary replacements, indicating that the presence of a foreign body activates IFN-gamma and causes CXCL10 to be produced, even in the absence of bacteria in the periprosthetic space (Figure 2c). IL-10 was also found at a higher concentration in both aseptic and septic revisions compared to primary replacement, suggesting the presence of an inflammatory milieu (Figure 2d). Taken together, these data indicate that the prosthesis is able to elicit an immune response despite its biocompatible nature, even in the absence of bacterial colonization. Understanding the immune reactions to prosthetic materials is crucial for ensuring their long-term compatibility with the body and minimizing complications or adverse effects.

### 2.2. Immune Response in Septic Revisions

**Figure 3:**
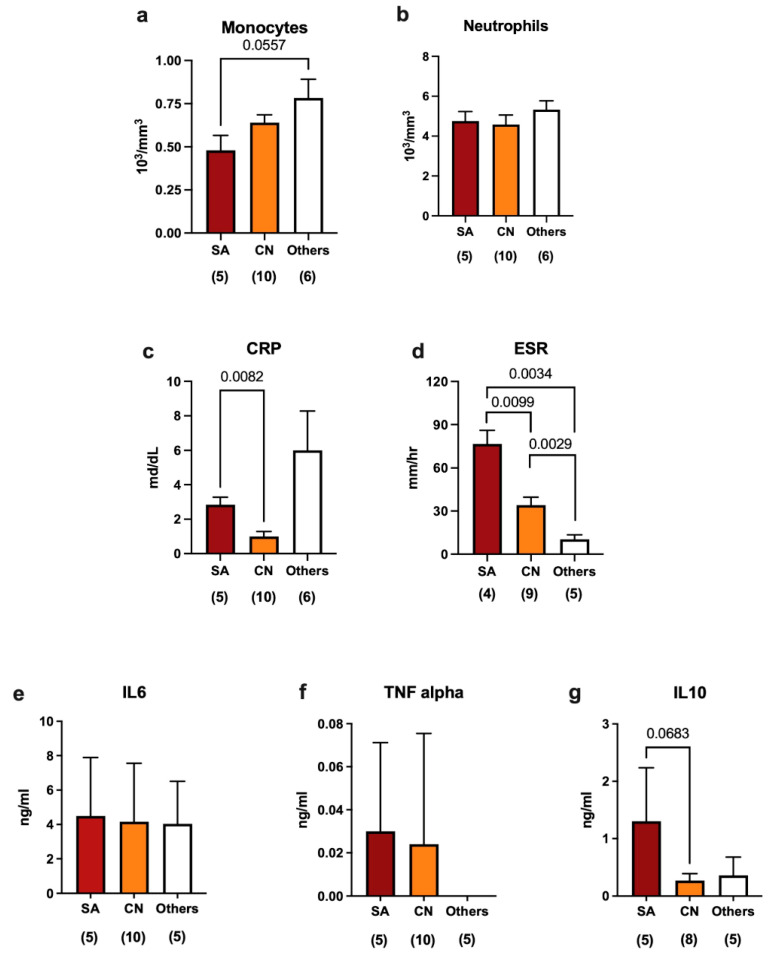
Immune modulation in response to different bacterial species in septic revisions. (**a**–**d**) Blood-circulating monocytes, neutrophils, C-reactive protein (CRP), and erythrocyte sedimentation rate (ESR) indicate the status of infection at systemic levels. (**e**–**g**) ELISA quantification of IL-6, TNF alpha, and IL-10 in the synovial fluids of patients with joints contaminated by different bacterial species. The number below each group represents the number of samples analyzed. Histograms represent the mean ± standard deviation. Statistical analysis: unpaired *t*-test with Welch’s correction.

In the peripheral blood samples we analyzed, high levels of monocytes and neutrophils (Figure 3a,b) were found regardless of the bacterium responsible for the infection. Instead, we observed a significant reduction in the production of the C-reactive protein (CRP), which is a marker of infection, in patients with synovial fluids contaminated by coagulase-negative bacteria (caused in eight cases by *S. epidermidis*, in one by *S. capitis*, and in one by *S. caprae*) compared to the case of *S. aureus* (Figure 3c). Moreover, differences in the erythrocyte sedimentation rate (ESR) between infections caused by *S. aureus* and those caused by other bacteria (*Streptococcus sanguinis*, *Enterococcus faecalis*, and *Streptococcus group C*) were observed (Figure 3d). Taken together, these data suggest that infections caused by coagulase-negative bacteria are characterized by a weaker immune response compared to those caused by both *S. aureus*.

In terms of the production of cytokines in synovial fluids, we observed that IL-6 was secreted at significant levels independently from the specific bacterium responsible for the infection (Figure 3e), which is expected since IL-6 is used as a marker of infection. TNF-alpha was detected at very low levels and showed no significant differences between the types of bacteria (Figure 3f), while IL-10 was higher in the case of infections caused by *S. aureus* than in those associated with CN bacteria (Figure 3g). Taken together, these data demonstrate that different immune responses can be dependent on the specific type of bacteria causing the infection. In particular, CN bacteria seem to play a role in stimulating the immune response that is different from what is observed in other infectious contexts.

We found that the presence of a foreign body induces the recruitment of leukocytes at the implant site with cytokine release, regardless of whether there is a bacterial infection. Therefore, we wondered whether the surface topography of the prosthesis could also play a role in stimulating the immune response and the type of bacterial colonization. To this end, we took advantage of a wider database [34] in order to evaluate differences—in terms of dominant bacterial contamination—between cemented prosthesis (i.e., having a smoother surface) and uncemented ones (i.e., having a rougher surface). In this analysis, we focused on the two bacterial species usually associated with PJI: *S. aureus* and *S. epidermidis*. We found a larger proportion of *S. epidermidis* contamination with respect to *S. aureus* in uncemented implants compared with cemented ones (Table 2). Albeit limited by the number of cases and additional effects related to the material of the prostheses, these data suggest that the surface topography of the plant could play a role in causing different types of infections, thus inducing a different inflammatory response.

### 2.3. Pathogenesis Mechanisms in Periprosthetic Joint Infections: An In Vitro Model

**Figure 4:**
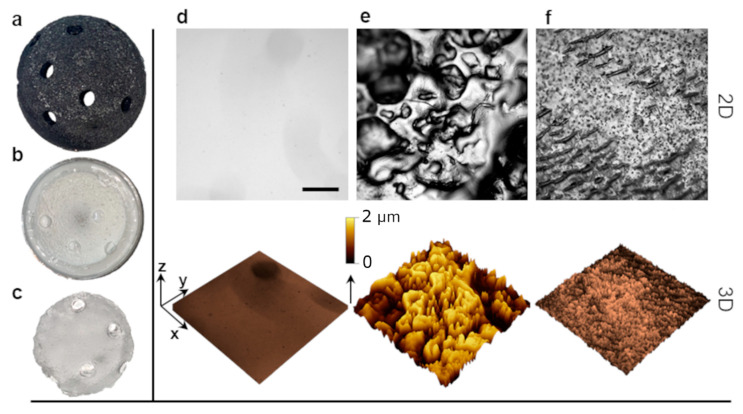
Fabrication of the PJI in vitro platform. (**a**) Commercial trabecular metal (TM) tantalum acetabular cup. (**b**) Negative replica of the cup in hard polyurethane binary resin. (**c**) Positive polydimethilsiloxane (PDMS) replica employed as a model substrate. (**d**–**f**) Surface characterization of different surface topographies. Phase contrast (PH) optical microscopy images and 3D topography projection of PDMS-replicated smooth and rough surfaces (**d**,**e**) compared with a cemented reference sample (**f**). Scale bar is 500 µm.

To probe the role of implant surface topography in PJI, we developed a replication protocol to obtain polydimethylsiloxane (PDMS) replicas of commercial trabecular metal (TM, Zimmer Biomet, Warsaw, IN, USA) acetabular cups (Figure 4a–c). Specifically, a negative mold of the tantalum acetabular cup was first obtained in a hard polyurethane binary resin (PU). The PU mold was then replicated in PDMS to obtain an exact positive replica of the original cup. This versatile two-stage replica-molding protocol allows for the large-scale production of rough surfaces bearing the exact topography of the original tantalum cup. Moreover, as PDMS is a soft elastomeric material, PDMS replicas can be cut in the shape of circular coupons to fit the bottom of a 96-well plate. The topographical analysis of the model surfaces was carried out by means of phase contrast optical microscopy (Figure 4d,e). For our in vitro platform, rough PDMS surfaces were compared to smooth analogues, obtained by casting a flat 2 mm PDMS film over a silicon wafer. We also analyzed the surface topography of a ‘cemented’ patch used as a reference for cemented implant surfaces.

To analyze the roughness of the PDMS surface replicas, 3D topography projections of the surfaces were extracted from the optical microscopy data after pixel mapping and calibration. Surface roughness is normally reported in terms of the arithmetical mean deviation of the profile $R_{a}=1/\Delta x\int_{x_{0}}^{x_{1}}z\left(x\right)dx\;$ or, alternatively, as the root-mean-square $R_{q}={\left(1/\Delta x\int_{x_{0}}^{x_{1}}z^{2}\left(x\right)dx\right)}^{1/2}$, whereas Āmax is defined as the average maximum profile amplitude and it is extracted from line profiles traced across different areas of the surface [35]. The roughness data extracted from the PDMS replica surfaces described in Figure 4d–f are reported in Table 3.

The results for smooth surfaces were in line with the literature data for native PDMS roughness [36]. For rough surfaces replicated from the TM acetabular cup, the estimated roughness is also comparable with the measurements reported in other works [37]. The roughness analysis proves the capability of this replica-molding process to consistently recreate the original implant topography in a model material easier to cast, transparent, and biocompatible, such as PDMS. Using this platform, biofilm formation and immune cell distribution could be monitored over time using epifluorescence microscopy and samples could be collected for the downstream analysis of inflammatory mediators release. Thanks to this in vitro model, we could investigate the sole effect of substrate topography on bacterial colonization and immune cells’ response, without changing other parameters of the surface, such as stiffness and chemistry.

### 2.4. Surface Topography Modulates Both Bacterial Growth and Immune Response

**Figure 5:**
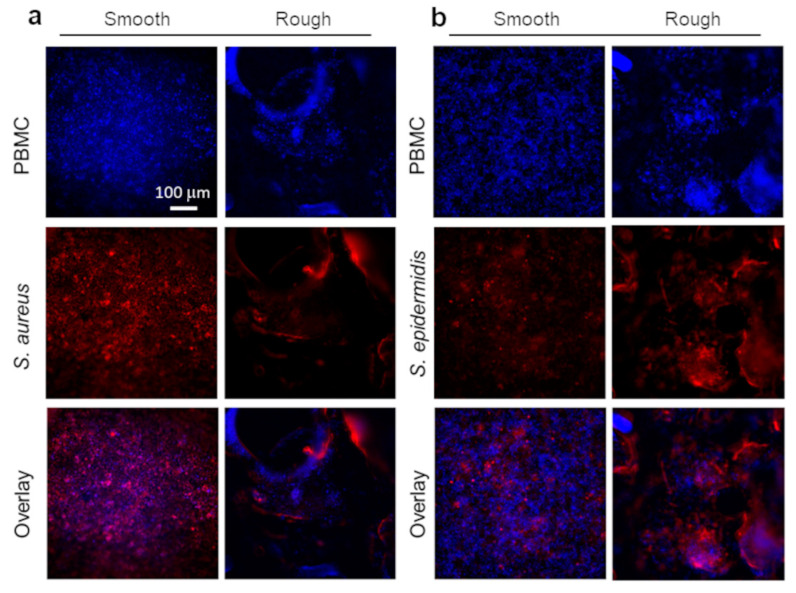
Fluorescence optical microscopy analysis for the in vitro model topographies in the presence of (**a**) PBMCs and *S. aureus* and (**b**) PBMCs and *S. epidermidis.* PBMCs were stained by means of CFSE CellTrace emitting at 517 nm (blue channel), whereas for *Staph.* spp., a CMPTX Cell Tracker emitting at 602 nm (red channel) was used. Panels (**a**,**b**) report single blue and red channels and an overlay of the two merged channels.

We initially exploited the in vitro platform to probe the sole bacterial interaction with the model surfaces for 24 h and 48 h. From fluorescence optical microscopy imaging, both *S. aureus* and *S. epidermidis* were able to effectively colonize both smooth and rough surfaces. Although, on rough surfaces, *S. aureus* biofilm was characterized by more segregated patches, *S. epidermidis* was able to uniformly colonize all surface portions. Subsequently, we tested the microbial colonization of the model surfaces in the presence of human peripheral mononuclear cells (PBMCs), retrieved from a panel of healthy donors. The epifluorescence imaging at 48 h is reported in Figure 5. PBMCs were stained with a blue fluorescent dye (CellTrace™ violet proliferation kit), while a red fluorescent dye (CellTracker™ Red CMTPX) was employed for *S. aureus* and *S. epidermidis*.

From imaging data, PBMCs were able to proliferate equally onto smooth and rough surfaces, although on rough surfaces, cell distribution was not homogenous, with clustering and segregation in the proximity of the negative curvature area regions (i.e., bottom of the pattern protrusions). From the overlays of the red and blue channels (Figure 5a,b), for *S. aureus* on rough surfaces, colocalization with PBMCs appears less evident, with two different populations of segregated cells and bacteria. On smooth surfaces, on the other hand, *S. aureus* is slightly prominent over the immune cells, meaning that *S. aureus* biofilm formation is favored on smooth surfaces for our experimental conditions. For *S. epidermidis*, instead, bacterial colonization is consistent all over both rough and smooth surfaces but with a higher amount of immune cells, due to the intrinsic low virulence of *S. epidermidis* compared to that of *S. aureus*.

**Figure 6:**
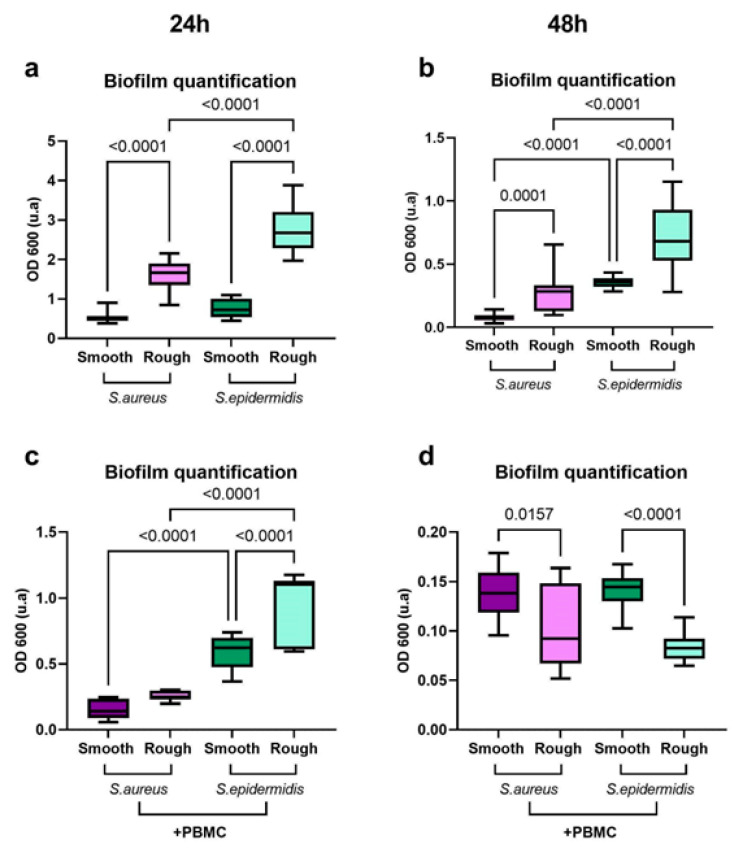
Biofilm quantification by crystal violet staining. (**a**,**b**) Biofilm quantification assay at 24 h (**a**) and 48 h (**b**) for *S. aureus* and *S. epidermidis* on smooth and rough surfaces. Both bacteria preferentially colonize rough surfaces, although, at 48 h, *S. epidermidis* biofilm production appears higher in general compared to that of *S. aureus* on both surfaces. (**c**,**d**) Biofilm quantification assay at 24 h and 48 h for *S. aureus* or *S. epidermidis* in the presence of PBMCs. At 24 h (**c**), bacterial growth is prominent and not influenced by immune cells. At 48 h (**d**), on rough surfaces, a reduction in biofilm production is observed indicating a response from immune cells to microbial proliferation. Statistical analysis: unpaired *t*-test with Welch’s correction.

To test the hypothesis that microbial colonization preferentially happens on rough surfaces and how the immune response can modulate such colonization, we performed a biofilm formation assay on the model surfaces with either *S. aureus* or *S. epidermidis* in the presence or absence of PBMCs. From the biofilm quantification assay conducted by the crystal violet staining of the biofilm [38] and the spectrophotometric recording of the optical density at 600 nm (OD 600), both bacterial species generally colonize more rough surfaces, with *S. epidermidis* almost doubling the amount of biofilm produced (Figure 6a,b). In the presence of PBMCs, at 24 h, bacterial proliferation prevails, but at 48 h, immune cells are able to counter bacterial infection, especially on rough surfaces (Figure 6c,d). In this case, the additional surface area given by the rough topography can be beneficial for the PBMCs, providing a stronger adhesion and the ability to better respond to microbial infections, although for both cells and bacteria, a reduction in vitality was observed.

**Figure 7:**
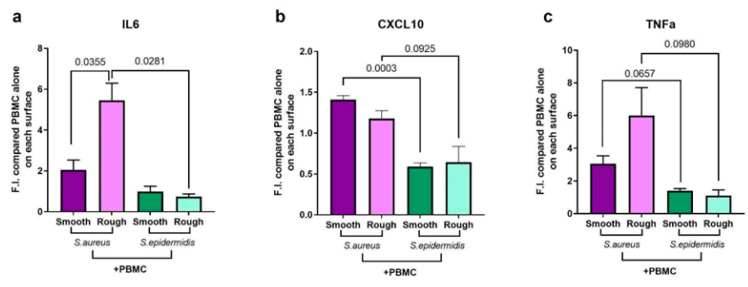
ELISA quantification in the co-culture of PBMCs and bacteria compared to the ELISA quantification of PBMCs cultured alone on different textured surfaces. We tested the release of (**a**) IL-6, (**b**) CXCL10, and (**c**) TNF-alpha in the supernatant. The columns represent the mean ± standard deviation of three independent donors. Statistical analysis: unpaired *t*-test with Welch’s correction.

Since we observed a vitality reduction, on the one hand, and reduced biofilm deposition, on the other, at 48 h, we decided to analyze the cytokines released by PBMCs placed on the biofilm formed by *S. aureus* and *S. epidermidis* at 24 h. As expected, *S. aureus* promotes inflammation, leading to the release of the inflammatory cytokines we analyzed (Figure 7, purple columns), although this production seems to be increased on rough surfaces in the case of IL-6 and TNF-alpha. In contrast, *S. epidermidis* is not able to elicit a strong inflammatory response (Figure 7, green columns), even when grown on rough surfaces. In fact, IL-6, CXCL10, and TNF-alpha were released at low levels from PBMCs in this infectious situation regardless of the surface of growth, thus indicating an inherent ability of the bacterium to evade the immune system.

## 3. Discussion

In this study, our initial objective was to investigate whether the prosthesis itself had the potential to attract leukocytes and trigger immune activation. When foreign materials are introduced into the body, the immune system typically responds by attempting to remove or isolate the foreign entity and prevent a chronic inflammatory response [21]. However, medical prostheses are generally designed to be biocompatible, meaning they are intended to minimize adverse immune reactions. Understanding the immune reactions to prosthetic materials is crucial for ensuring their long-term compatibility with the body and minimizing complications or adverse effects. For example, the safety of breast implants has been recently studied with respect to the occurrence of an anaplastic large cell lymphoma (ALCL) associated with the different surface texturizations [39]. Lymphomas have been correlated with other implantable devices, such as cardiac, gluteal, testicular, and intraocular implants [40,41]. In addition, implants may wear and the formation of debris can stimulate the immune system, leading to chronic inflammation and eventually to tumor formation [42,43].

We observed that the presence of a prosthesis, even in cases without evidence of infection, triggers immune responses characterized by the activation of T-helper and cytotoxic lymphocytes. T-helper lymphocytes play a critical role in coordinating immune responses by releasing cytokines, which in turn help to regulate and activate other immune cells involved in the defense against pathogens or foreign materials. Cytotoxic lymphocytes are responsible for directly targeting and eliminating infected or abnormal cells in the body. This immune activation suggests that the body recognizes the prosthesis as a foreign object, initiating an immune response to eliminate or control it. Further investigations and experiments may be needed to understand the underlying mechanisms of this immune response and its implications for prosthetic function, long-term biocompatibility, and patient outcomes. It also highlights the importance of considering the immune system’s response when developing and evaluating medical prostheses to ensure optimal biocompatibility and minimize potential complications. Prosthetic function refers to how well the prosthetic joint performs and whether it can effectively replace the function of the natural joint it is replacing. This involves assessing the prosthesis’s durability, stability, and range of motion, as well as evaluating the patient’s ability to perform daily activities and tasks with the prosthesis [44]. As a result, researchers are continually seeking new ways to prevent or identify infections early. This involves evaluating different materials and coatings for prostheses that may reduce the risk of infection, as well as developing new diagnostic techniques to identify infections early and allow for prompt treatment. In this work, we found that the leukocyte profile in the synovial fluid of aseptic revisions differed significantly from that of septic revisions. In septic revisions, there was a predominance of neutrophils; conversely, other leukocyte populations were almost entirely absent. This finding is expected because the innate immune system, particularly neutrophils, plays a crucial role in the immediate response to bacterial invasion [45]. The absence of other leukocyte populations in septic revisions suggests that the body’s immune system is primarily focusing its response on combating the infection. The differences in leukocyte profiles were further supported by the presence of pro- and anti-inflammatory cytokines in the synovial fluid. Both septic and aseptic revisions had higher levels of cytokine release compared to that of synovial fluids obtained during primary replacement surgeries. Specifically, IL-6, which is commonly used as a marker of infection, showed significantly higher levels in septic samples, as expected. However, our analysis also revealed that CXCL10 and IL-10 exhibited a similar trend. These findings suggest that CXCL10 and IL-10 could potentially serve as biomarkers to differentiate between septic and aseptic revisions, including cases where aseptic revisions are later reclassified as septic following the microbiological culture of the prosthesis or synovial fluid. By considering the release patterns of these cytokines, it may be possible to more accurately diagnose PJIs, especially in cases where infections are initially classified as aseptic but later found to be septic. Further research and validation studies would be necessary to confirm the utility of CXCL10 and IL-10 as additional biomarkers for PJI identification and to determine their effectiveness in clinical practice.

**Figure 8:**
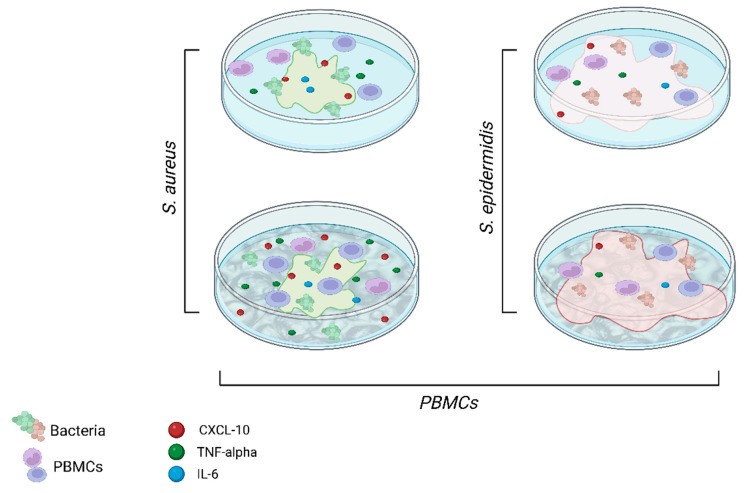
Schematic illustration summarizing the key features from the in vitro experimental findings. Created with BioRender.com.

This study indicated that cytokine release and the degree of inflammation are influenced not only by the presence of bacterial contamination but also by the specific type of bacterium involved and the type of prosthetic surface (as summarized in Figure 8). It has been observed how bacteria confinement could be induced providing surfaces with topographies commensurable to bacterial size [35], thus influencing bacterial behavior and distribution. For instance, trabecular metal with its void distribution can provide an additional surface area that can entrap bacteria. However, it is important to note that the two bacterial species predominantly associated with PJI, i.e., *S. epidermidis* and *S. aureus*, occupy these surfaces differently. *S. aureus* tends to exhibit clustering and segregation, resulting in less biofilm formation. On the other hand, *S. epidermidis* shows higher levels of biofilm production on these surfaces. These findings highlight the complexity of bacterial interactions with different surfaces and how it can influence their behavior, including their ability to form biofilms. The ability of *S. epidermidis* to form biofilms more effectively suggests that it may have a greater resilience and ability to persist on surfaces, potentially contributing to its ability to cause chronic infections. In addition, our results regarding cytokine responses further support the notion that *S. epidermidis* has a greater ability to evade immune detection independently of the surface it comes into contact with. On the other hand, *S. aureus* appears to exhibit different reactions depending on the specific contact surface it encounters, with a greater release of IL-6 and TNF-alpha in response to a rough surface. Therefore, understanding the interaction between *S. aureus* and porous implants, such as trabecular metals, is crucial in the development of biomaterials that minimize bacterial adhesion and promote a balanced immune response. It underscores the importance of considering not only the type of bacteria but also the characteristics of the prosthetic surface in developing effective preventive measures and treatment strategies.

Finally, we present a novel method to replicate the surface topography of prosthetic implants, which can be instrumental in the study of the interplay between biofilm formation, immune cells, and implant surface textures. This method paves the way for the development of innovative prosthetics with improved performance in terms of bacterial colonization and immune response. In our future work, we intend to investigate the relationship between the immune response and different levels of infectious loads. By studying the immune response to infections of varying severity, we can gain a better understanding of how the immune system reacts and adapts to different levels of bacterial presence. Moreover, it would be valuable to expand the in vitro platform by incorporating mechanical stress on various surface topographies. By subjecting the prosthetic surfaces to mechanical stress, we can simulate the wear and tear that occurs in real-life scenarios, which allow us to investigate the specific impact of friction debris on immune activation. This can provide insights into how the interaction between surface topography, mechanical stress, and immune cells influences inflammatory responses. All this information has the potential to enhance our understanding of immune responses in infections and contribute to the development of more effective strategies for managing and treating infections of varying severity.

## 4. Materials and Methods

### 4.1. Patients

We conducted an ex vivo study on samples of synovial fluid derived from patients undergoing primary joint replacement or revision surgery. Synovial fluids were collected from patients at the time of the surgery under complete sterile conditions. A subset of these samples, with sufficient quantity and absence of contamination, was subjected to FACS analysis. All patients were provided with written informed consent, which was signed before the surgery.

### 4.2. Flow Cytometry Experiments

Flow cytometry experiments were performed following standard procedures [46]. For the ex vivo analysis: CD45 PerCP Cy 5.5 (BD Biosciences, San Diego, CA, USA; catalogue number 564105); CD11b BUV805 (BD Biosciences, Eysins, Switzerland; catalogue number 748587); CD3 BUV395 (BD Biosciences, catalogue number 564001); CD19 APC (BD Biosciences, catalogue number 555415); CD4 BV421 (BD Biosciences, catalogue number 562424); CD8 FITC (BD Biosciences, catalogue number 555366); CD69 PeCy7 (BD Biosciences, catalogue number 557745); CD16 BV786 (Biolegend, San Deigo, CA, USA; catalogue number 302046); CD14 APC (BD Biosciences, catalogue number 555399); CD66b PeCy7 (Biolegend; catalogue number 305116); HLA-DR BV711 (BD Biosciences, catalogue number 563696); CD15 BUV496 (BD Biosciences, catalogue number 741187); CD33 BUV563 (BD Biosciences, catalogue number 741369); CD206 FITC (BD Biosciences, catalogue number 551135); CD163 PECF594 (BD Biosciences; catalogue number 568206); CD66b PECy7 (Biolegend Campoverde, catalogue number 305116); CD56 PE (BD Biosciences, catalogue number 555516); CD1a BV480 (BD Biosciences, catalogue number 565652); and CD4 BV421 (BD Biosciences, catalogue number 562424). Dead cells were excluded through Live and Dead (BV510) staining (Invitrogen catalogue number L34957). Labeled cells were fixed in PBS + 1% formalin. The acquisition was performed at FACSymphony A5 (BD Biosciences) and analyzed by FACS Diva and FlowJo software version 6.1.1 (BD Biosciences). For the CD45 count, a known amount of synovial/peri-prosthetic fluid was labelled with CD45 PerCP Cy 5.5 (BD Biosciences) and added to the labelled cells, and washed with FACS buffer in a tube containing labelled marbles of known quantity/μL (BD Leucocount™ Kit, BD Biosciences, San Diego, CA, USA; catalogue number 340523). Our lot contained 56150 beads for each tube (BD Leucocount™ Kit, BD Biosciences, San Diego, CA, USA; catalogue number 340523). Acquisition was performed at FACS Symphony. The number of CD45/μL was estimated as: no. CD45 counted/no. beads counted x total beads/μL marked.

### 4.3. Model Surface Preparation

Textured surfaces for the in vitro model were prepared by the replica-molding of commercial tantalum acetabular cups (Trabecular Metal Revision Shell, ZimmerBiomet). Replication consisted in a two-step procedure where the cup was initially molded in a polyurethane binary resin (Smooth-cast 300, 1:1 part A:part B) in a Petri dish and cured on a hot plate for 30 min at 65 °C. The PU negative mold was then replicated in polydimethylsiloxane (Sylgard 184, Dow Corning, Midland, MI, USA) with a curing ratio of 10:1 prepolymer to curing agent. The mixture was vigorously stirred and placed in a desiccator to remove air inclusions for 10 min. The mixture was then cast in the PU mold and cured on a hot plate at 75 °C for 1 h. Once cured, the PDMS-positive replica was peeled from the PU mold and the surface roughness and morphology were analyzed by means of phase-contrast optical microscopy (Leica DMi8). Roughness analysis and 3D topography maps were extracted after the grayscale mapping calibration of the optical images.

### 4.4. Cell Culture Experiments

Human peripheral blood mononuclear cells (PBMCs), derived from healthy donors’ buffy coats, were isolated by density gradient centrifugation on Lympholyte^®^-H Cell Separation Media (Euroclone, Lima, Peru). PBMCs were stained with 2.5 mM of CellTrace™ Violet Cell Proliferation Kit (Invitrogen) for 7 min at 37 °C, and then washed with a double volume of cold FBS (Lonza, Bend, OR, USA) and Roswell Park Memorial Institute (RPMI) 1640 Complete Medium (Lonza) consisting of RPMI supplemented with 10% FBS (Lonza), 1% Penicillin–Streptomycin (Invitrogen, Carlsbad, CA, USA), and 1% Ultra Glutamine (Lonza). Stained cells were left at 4 °C for 1 h and successively centrifuged and resuspended in RPMI 1640 medium supplemented with 10% FBS and 1% Ultra Glutamine without antibiotics. They were seeded at a concentration of 2 × 10^6^ cells in 24-well microplates (COSTAR, 13 Corning Incorporated, Brooklyn, NY, USA). Cells were then cultured on different model surface textures for 48 h, at 37 °C and 5% CO_2_.

### 4.5. Bacterial Cultures

*S. aureus* (ATCC 6538, Sigma Aldrich, Saint Louis, MO, USA) and *S. epidermidis* (ATCC 12228, Sigma Aldrich) were plated on agar plates (Columbia agar with 5% sheep blood, BD) and incubated at 37 °C overnight. Single colonies were scraped and suspended in 2 mL sterilized rich medium (Bacto Tryptone 15g/L, BD) for 2 h. Bacterial suspensions were then stained using a CMPTX red dye (Cell Tracker red CMPTX dye, ThermoFisher, Waltham, MA, USA). Specifically, a 10 mM stock solution of the dye was prepared diluting the lyophilized product with dimethyl sulfoxide (DMSO, HPLC grade, 99.9+%, Thermoscientific, Waltham, MA, USA). The stock solution was then diluted to a working concentration of 2 mM in the final bacterial suspension volume. The suspension was then vortexed and incubated for 30 min at 37 °C. After incubation, the suspension was centrifuged at 4000 rpm for 15 min to recover the stained bacteria. Finally, the supernatant was discarded and the bacterial cells were suspended in RPMI medium without antibiotics.

### 4.6. In Vitro Model Platform Preparation

For the preparation for the in vitro model platform, 8 mm circular coupons were cut from the smooth and rough replica samples using a histology punch and attached with a drop of uncured PDMS to the bottom of a 24-well or 96-well culture plate (COSTAR, 13 Corning Incorporated, NY 14831, USA). This procedure prevented PDMS coupons from floating once the cells and bacteria suspensions were inoculated. The well plate was then placed on a hot plate at 60 °C for 30 min to allow for PDMS to cure. The in vitro platform was then UV sterilized for 30 min prior to cell inoculation. The well plate was designed, for tests with bacteria only, with two columns (three coupons each) dedicated to *S. aureus* on smooth and rough surfaces and two columns dedicated to *S. epidermidis* on smooth and rough surfaces alongside control columns without bacteria. In tests with PBMCs and bacteria, two columns were dedicated to PBMCs on smooth and rough model surfaces. Firstly, 500 μL of the bacterial suspensions (optical density OD600 nm = 0.2) were incubated in the respective wells. Afterwards, 500 μL of the PBMC suspension at a concentration of 2 × 106 cells were added, and the plate and incubated at 37 °C with 5% CO_2_ for 24 h or 48 h. At the given time point, PBMCs and bacteria co-cultures on the different model surfaces were imaged by fluorescence optical microscopy.

### 4.7. Diagnostic Assay (ELISA)

Supernatant fluids from cell cultures were collected after 24 or 48 h of cell culture and treated in the centrifuge at 1200 rpm for 5 min. Fluids collected from the periprosthetic space were also treated in the centrifuge at 1200 rpm for 5 min. To quantify the production of human IL-6, TNF-alpha, CXCL10, and IL-10 in cell supernatants and synovial fluids, commercial ELISA kits were used following the manufacturer’s instructions (R&D Systems, Minneapolis, MN, USA). Data were analyzed with SoftMax Pro 5.3 software.

### 4.8. Biofilm Growth Assay (Crystal Violet)

Supernatant fluids from bacteria and cell cultures were removed and each well rinsed twice with sterile distilled water and dried at 37 °C. A total of 300 μL of 1% crystal violet (CV, 90% anhydrous Merck, Rahway, NJ, USA) in ethanol (absolute, ACS grade, Merck) were added to each well and incubated at room temperature for 15 min. The CV was then removed and each well rinsed three times with distilled water and the plate dried for 15 min. The CV was then dissolved by adding 1 mL of 30% acetic acid (ACS grade 99.7%, Merck) and the supernatant of each well was transferred in a fresh 96-well plate to measure the optical density of CV at 600 nm (OD600).

### 4.9. Statistical Analysis

Statistical analysis was performed using an unpaired *t*-test with Welch’s correction. Continuous variables and all data were described using mean ± SD. A *p*-value of less than 0.05 was considered statistically significant.

## Figures and Tables

**Table 1:** Patients’ baseline characteristics.

Number of patients	74
Septic revision	50
Aseptic revision	18
Primary replacement	6
Age median (IQR)	63 (33.5–90.3)
Septic revision	71 (33.5–90.3)
Aseptic revision	64 (53–86)
Primary replacement	64.5 (57–84)
Sex (female)	29 (39%)
Septic revision	18
Aseptic revision	10
Primary replacement	1
Hip	61 (82%)
Septic revision	31
Aseptic revision	15
Primary replacement	5
Knee	13 (18%)
Septic revision	10
Aseptic revision	2
Primary replacement	1
Etiology	
*Staphylococcus aureus*	6 (14%) ^1^
Coagulase Negative	11 (26%) ^1^
*Staphylococcus epidermidis*	7 (17%) ^1^
Others	25 (60%) ^1^

^1^ Percentages expressed taking the total number of infected patients as the reference.

**Table 2:** Prevalence of *S. aureus* (SA) and *S. epidermidis* (SE) among septic partial or total one-stage revisions performed at Humanitas Research Hospital in the period of 2016–2019 [27].

	Total SR	SA	SE	SE/SA
Cemented	25	3	6	2
Uncemented	111	8	22	2.75

**Table 3:** Roughness average (Ra), root-mean-square roughness (Rq), and average maximum amplitude (*Ā_max_*) of the in vitro model topographies reported in Figure 5. Ra, Rq, and *Ā_max_* were averaged over a series of 10 line profiles extracted from 3 different images per sample type.

	Ra (µm)	Rq (µm)	Āmax (µm)
Smooth	0.025 ± 0.008	0.042 ± 0.008	0.090 ± 0.010
Rough	2.240 ± 0.070	2.980 ± 0.070	3100 ± 0.095
Cement	0.110 ± 0.020	0.150 ± 0.020	0.250 ± 0.050

## Data Availability

The data presented in this study are openly available in Zenodo at 10.5281/zenodo.7950576.
